# Seed characteristic variations and genetic structure of wild *Zizania latifolia* along a latitudinal gradient in China: implications for neo-domestication as a grain crop

**DOI:** 10.1093/aobpla/ply072

**Published:** 2018-12-02

**Authors:** Yao Zhao, Lan Zhong, Kai Zhou, Zhiping Song, Jiakuan Chen, Jun Rong

**Affiliations:** 1 Jiangxi Province Key Laboratory of Watershed Ecosystem Change and Biodiversity, Center for Watershed Ecology, Institute of Life Science and School of Life Sciences, Nanchang University, Nanchang, China; 2 The Ministry of Education Key Laboratory for Biodiversity Science and Ecological Engineering, Institute of Biodiversity Science, Fudan University, Shanghai, China; 3 Institute of Vegetable, Wuhan Academy of Agriculture Science and Technology, Wuhan, Hubei Province, China

**Keywords:** domestication, genetic structure, grain crop, seed characteristics, *Zizania latifolia*

## Abstract

Crop wild relatives are not only important genetic resources for crop improvement, but also domestication candidates for selecting new crops. As a close relative of American wild rice *Zizania palustris*, *Z. latifolia* is a perennial aquatic grass widely distributed in China. Although *Z. latifolia* has been domesticated and cultivated as an aquatic vegetable for >1000 years, a neo-domestication for grain production needs to be soundly evaluated. In this study, we investigated the seed characteristic variations and genetic structure of 15 *Z. latifolia* wild populations along a latitudinal gradient in China. Our results showed that *Z. latifolia* tended to produce relatively larger seeds with lower moisture content and lower investments in seed pericarp at lower latitudes. The width, size, shape, seed-pericarp ratio and relative water content of seeds were significantly associated with climatic variables. The seeds of *Z. latifolia* showed a relatively low germination percentage and strong dormancy, which might hinder the neo-domestication. In addition, high genetic differentiation had been found among *Z. latifolia* populations, which could be attributed to isolation by distance. This study offered preliminary information for the utilization and conservation of wild *Z. latifolia*. It suggested that the wild populations in the middle and lower reaches of the Yangtze River could be good candidates for grain crop domestication due to appropriate seed traits and high genetic diversity. The neo-domestication of wild *Z. latifolia* requires further researches on the genetic mechanism of the Domestication Syndrome and more works on artificial breeding.

## Teaser

*Zizania latifolia* is a perennial aquatic grass that has been domesticated as a vegetable in China. As a close relative of American wild rice *Z. palustris*, this species holds great potential in grain production. We found that *Z. latifolia* tended to produce larger seeds with lower moisture content and less investments in seed pericarp at lower latitudes. This suggested that the populations in the middle and lower reaches of the Yangtze River could be good candidates for grain crop domestication due to appropriate seed traits and high genetic diversity.

## Introduction

The growing global human population results in increasing demands on cultivated plant resources (FAO 2014). Cultivated plants were domesticated from their wild relatives. Wild relatives of cultivated plants have accumulated more abundant genetic and morphological variations during long-time natural evolution, which can either be used as genetic donors for the improvements of cultivated plants or domestication candidates for selecting new crops (Hajjar and Hodgkin 2007). New crops could provide a wide array of benefits to farmers, consumers and the environment (Janick *et al.* 1996; DeHaan *et al.* 2016). For example, a new perennial grain crop with an extraordinary performance on biomass accumulation would not only have high impacts on the production mode for current annual crops (Cox *et al.* 2006; Glover *et al.* 2010; Brummer *et al.* 2011), but also could be used for forage or biofuel (Leakey 2007; Sang 2011). In the past 50 years, the list of domesticated crops has been revised to include many more species (Meyer *et al.* 2012). Most of these newly domesticated crops were selected from wild relatives of cultivated plants owing to the translational researches of popular crops (Hajjar and Hodgkin 2007; Ronald 2014; Jacob *et al.* 2017). Although domestication of new crops is inspiring, it requires explicit studies on morphological and genetic variations of the domestication candidate under a theoretical context (Diamond 2002; Runck *et al.* 2014; DeHaan *et al.* 2016).

The genus *Zizania* is a member of tribe Oryzeae in Poaceae. This genus consists of four species, including *Zizania aquatica*, *Zizania palustris*, *Zizania texana* and *Zizania latifolia* (Terrell *et al.* 1997; Chen *et al.* 2012). The first three are naturally distributed in North America, whereas *Z. latifolia* is native to East Asia (Xu *et al.* 2010; Chen *et al.* 2012). The American ‘wild rice’ *Z. palustris* has been harvested for seeds by Native Americans for centuries. Domestication of this species began in the first half of the 20th century (Oelke 1993). The scientists from University of Minnesota played an important role in the wild rice domestication and breeding, and their efforts led to genetic and agronomic advances. The wild rice is now becoming an established grain crop in North America (Oelke 1993). On the other hand, as a relative of the American wild rice, *Z. latifolia* was once one of the important grain plants in ancient China from the Zhou to the Tang Dynasty (771 B.C. to 907 A.D.) (Guo *et al.* 2007). However, *Z. latifolia* has been cultivated as a popular aquatic vegetable for >1000 years because the young shoots of the plant become swollen and edible after being infected by the fungus *Ustilago esculenta* (Guo *et al.* 2007; Xu *et al.* 2008). Although *Z. latifolia* eventually missed the chance to be domesticated as a grain crop in history, it is proved to be a valuable germplasm resource for the improvement of Asian cultivated rice (*Oryza sativa*) varieties (Chen *et al.* 2006). The success of American wild rice domestication and the elite agronomical traits found in *Z. latifolia* together suggest that it is possible to domesticate *Z. latifolia* to a new grain crop.

*Zizania latifolia* is a perennial emergent macrophyte widely distributed in the wetlands across eastern China. This plant has an outcrossing mating system and well-developed rhizomes, and it can reproduce both sexually and asexually through seeds, rhizomes and tiller buds (Guo *et al.* 2007). It is a pioneer species with a high morphological plasticity and tolerance to submergence, heavy metal ion pollution and eutrophication (Xu *et al.* 2008). This species is important not only because of its ecological functions but also as a type of agricultural resource. Cultivated *Z. latifolia* has been widely planted and now is only second to the lotus (*Nelumbo nucifera*) among the main aquatic vegetables cultivated in China (Guo *et al.* 2007; Chen *et al.* 2012). Many previous studies have been conducted on cultivated *Z. latifolia*, including its systematic position, utilization as the tertiary gene pool of rice, nutritional value, cultivar classification and breeding (Chen *et al.* 2006; Guo *et al.* 2007; Xu *et al.* 2008, 2010). However, we still lack comprehensive understandings on the morphological and genetic variations in wild *Z. latifolia* populations, which are indispensable to evaluate the domestication potential (Chen *et al.* 2012; Fan *et al.* 2016).

Because the seed traits are important for grain crops, they should be firstly characterized before the neo-domestication. For example, seed size, seed dispersal, seed longevity and dormancy are dramatically divergent between cultivated grain crops and their ancestors (Doebley *et al.* 2006; Gross and Olsen 2010). The archaeological evidence had also indicated that the seed traits were the direct targets under artificial selection in the historical domestication of grain crops (Purugganan and Fuller 2009). In this study, we investigated the variations in seed characteristics (mainly including seed size, germination percentage, dormancy, relative water content and seed-pericarp ratio) and genetic structure for 15 *Z. latifolia* wild populations sampled along a latitudinal gradient in China. With these surveys, we attempted to address: (i) the pattern of the variance in seed characteristics among populations and their correlations with climatic variables; (ii) the baseline morphological and genetic information for the neo-domestication of *Z. latifolia*.

## Materials and Methods

### Sample collection

**Figure 1.**
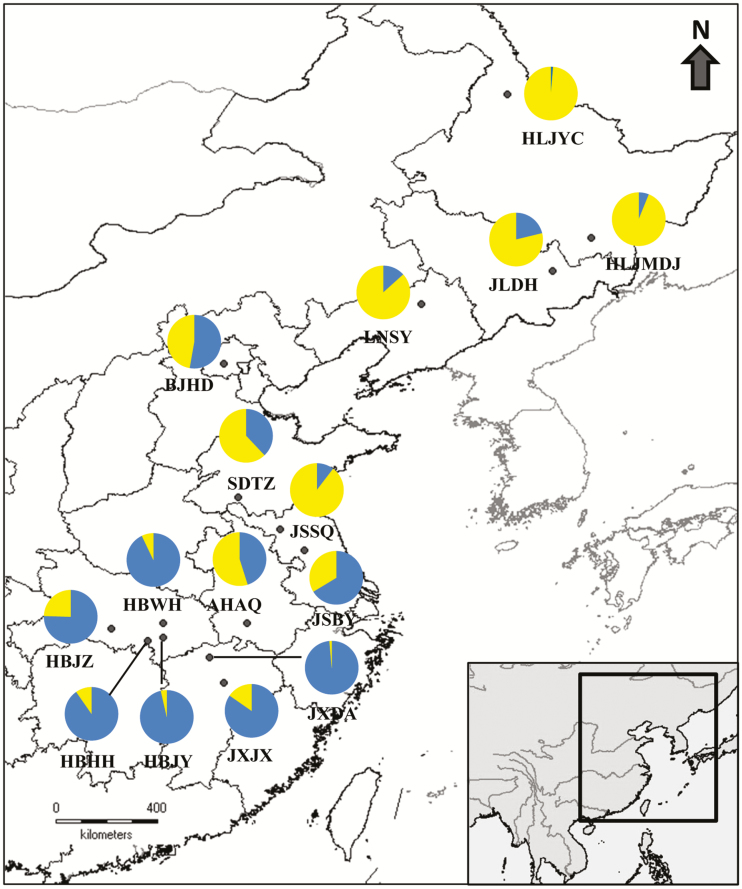
Geographic locations of the sampling sites and corresponding genetic assignments (pie charts) by STRUCTURE for the 15 *Zizania latifolia* populations in China.

We conducted a north-to-south transect along a latitudinal gradient in China and sampled 15 *Z. latifolia* populations (Fig. 1 and **see****Supporting Information—Table S1**). For each population, the grains (caryopses) were collected from the fruiting plants and pooled. A few grains were chosen to investigate the variations in seed characteristics, and the remaining grains were stored in moist sands and kept at 4 °C to simulate the natural conditions for germination experiments. To characterize the genetic diversity and structure of *Z. latifolia* populations, ~30 leaf samples were randomly collected in each population with an interval of ~5 m between adjacent plants for DNA extraction. Fresh young leaves were collected and placed in a plastic bag containing silica gel.

### Habitat heterogeneity

The geographic information for each sampling site was collected using a GPS navigator upon sampling. The environmental layers of 19 bioclimatic variables (bio1–19) and monthly mean temperature and precipitation (1950–2000) were obtained from the WorldClim version 2.0 website (Hijmans *et al.* 2005; http://www.worldclim.org). The resolution of all climatic layers was 2.5 arc-minutes (5 km), to be compatible with the resolution of the locality data. In addition, we generated the water availability and temperature variability at the time of seed released from the plants based on the monthly mean temperature and precipitation, including the Max Temperature of Seed dispersal Month (bio20), Min Temperature of Seed dispersal Month (bio21) and Mean Precipitation of Seed dispersal Month (bio22). Thus, we included all 22 climatic variables to infer the habitat heterogeneity. Given the high degree of cross-correlation between these climatic variables, the principal component analysis (PCA) was used to reduce the dimensionality of the climatic data set.

### Trait measurements and analysis

Firstly, 100 high-quality grains (plump grains with undamaged awns and hulls) were randomly chosen from each population for trait measurements. The grain length (*GL*), seed (hulled grain) length (*SL*) and seed width (*SW*) were measured with a digital caliper. Because the awn is an important accessory structure of seed and can affect the activity of seed dispersal, the awn length (*AL*) was also measured. From these measurements, the seed size (*S*_size_ = *SL* × *SW*), seed shape (*S*_shape_ = *SL*/*SW*) and awn-length/grain-length ratio (*AGL*) were calculated. Then, another 100 grains were randomly selected from the mixed seed pool of each population. They were oven-dried and weighed (*M*_100grain_). Then, they were hulled and weighed to obtain the weight of 100 seeds (*M*_100seed_). This procedure was repeated three times to avoid biases, and the mean value was recorded.

In addition, the relative water content (*WC*) and seed-pericarp ratio (*SPR*) were investigated. For each population, 100 mature grains were fully hydrated and weighed (*M*_0_). They were oven-dried for 5 days to a constant weight and weighed again (*M*_D_). The *WC* was then estimated [*WC* = (*M*_0_*− M*_D_)/*M*_D_]. The hulls (the pericarps of caryopses) of these dehydrated grains were removed, and the seeds were weighed (*M*_N_). The *SPR* was simply the seed pericarp proportional to dry seed mass [*SPR* = (*M*_D_*− M*_N_)/*M*_D_]. These procedures were repeated three times to obtain an average value.

As described by Kovach and Bradford (1992), the grains were stored in moist sands at 4 °C for 6 months of stratification. Before germination, the awns and hulls of grains were removed to break dormancy. The seeds were treated with a 0.1 % HgCl_2_ (w/v) solution for 5 min and washed five times with distilled water. One hundred treated seeds were chosen from each population and put in a Petri dish containing distilled water for germination, with two replicates per population. The seeds were germinated at 24 °C with a 12-h/12-h (light/dark) photoperiod. Germination was monitored for 30 days, and the number of germinated seeds was recorded each day. The germination percentage for each population was determined as a proportion of the initial number of seeds. A tetrazolium test was performed on the seeds that failed to germinate after 30 days to check their viability. The seeds were cut longitudinally through the embryo, soaked in a 0.15 % (w/v) 2,3,5-triphenyltetrazolium chloride solution for 20 h at 20 °C in the dark, and scored according to the intensity and location of staining.

A descriptive analysis was performed to characterize the variations of the measured seed traits using SPSS v22.0 (IBM Corp. 2013). One-way ANOVAs were then applied to analyse the significance of the differences between the means of these traits, with population as the main effect. Subsequent pairwise contrasts were performed with the least significant difference (LSD) tests, which were considered significant at *P* < 0.05. Principal component analysis was performed to reduce the data set to sets of interrelated variables. Correlation analyses were performed using SPSS v22.0. Multiple regressions were also applied to investigate the associations between the variations of the seed characteristics and climatic variables.

### Genetic variations and clonality estimation

#### DNA extraction and genotyping

We extracted DNA and amplified eight highly polymorphic microsatellite loci (screened from 16 species-specific microsatellite markers) for each sample following the methods described in Quan *et al.* (2009). The detailed information of microsatellite markers was shown in **Supporting Information—Table S6**. The PCR products were labelled using Fam-, Tamra- and Hex-labelled primers. Alleles were sequenced on an ABI 3730 (ABI) automated sequencer using LIZ 500 as a ladder and analysed in Genemapper v4.0 (ABI).

#### Clonality and genetic structure

Because of the intensive clonal reproduction of *Z. latifolia*, we assumed that the different occurrences of the same multilocus genotype (MLG) within a population were ramets (clonemates). Therefore, a single representative of each MLG (genet) was retained to analyse for genetic variations.

Genets were identified using similarity thresholds based on the frequency distribution of pairwise genetic distances between ramets (MLGs) (Douhovnikoff and Dodd 2003) as implemented in GENOTYPE (Meirmans and van Tienderen 2004). A threshold should be chosen to assign each ramet to the same multilocus lineage (MLL, genet). The introduction of MLL was used to reveal the existence of somatic mutations or scoring errors in the data set resulting in low distances among slightly distinct MLG actually deriving from a single reproductive event (Arnaud-Haond *et al.* 2007). The appropriate threshold was chosen following Douhovnikoff and Dodd (2003) based on the distribution histogram of Dice similarity. The existence of clonal growth would induce MLG pairs presenting extremely low distance, and originate a primary small peak in the frequency distribution of distances, making it bimodal rather than unimodal (Arnaud-Haond *et al.* 2007). We drew the frequency distribution of the values of all comparisons **[see****Supporting Information—Fig. S1****]**. The valley between the first and second peaks was considered a good candidate to use as a threshold (Meirmans and van Tienderen 2004). Therefore, we set the threshold = 1. The number of genets (*G*), Simpson’s diversity index (*D*) and evenness (*E*) were calculated by GENODIVE (Meirmans and van Tienderen 2004). The clonal richness index was calculated as *R* = (*G* − 1)/(*N* − 1), where *N* is the number of ramets.

MicroChecker (van Oosterhout *et al.* 2004) was applied, and no significant evidence was found for the presence of null alleles (the estimated null allele frequency < 0.05; **see****Supporting Information—Table S6**). GenAlEx v6.5 (Peakall and Smouse 2012) was used to compute the parameters of genetic variations based on the genotypic data of genets. We tested the departures from Hardy–Weinberg equilibrium and heterozygote deficiency/excess and calculated the population fixation index values (*F*_is_). The global and pairwise differentiation coefficients *F*_st_ were estimated. Partitioning of the total genetic variation within and between populations was further analysed by analysis of molecular variance (AMOVA) with 1000 permutations. The Mantel test was applied to detect the effect of isolation by distance (IBD) on the population structure. The pairwise geographical distance (*GGD*) matrix and genetic distance [pairwise *F*_st_/(1 *− F*_st_)] were generated, and the Mantel test was performed with 1000 bootstraps.

The genetic structure was further explored using the Bayesian clustering algorithm implemented in STRUCTURE 2.3.4 (Pritchard *et al.* 2000). The program was given no prior information on ancestral populations and run 10 times for each value of *K* (1–15) ancestral populations under the admixture model with correlated allele frequencies, using 200 000 Markov chain Monte Carlo iterations and a burn-in of 100 000 iterations. The optimal number of genetic clusters was determined using Δ*K* (Evanno *et al.* 2005). The resulting matrices of estimated cluster membership coefficients (*Q*) were permuted with CLUMPP (Jakobsson and Rosenberg 2007). The final matrix for each *K* value was visualized with DISTRUCT (Rosenberg 2004).

## Results

### Climatic heterogeneity of the sampling sites

**Figure 2.**
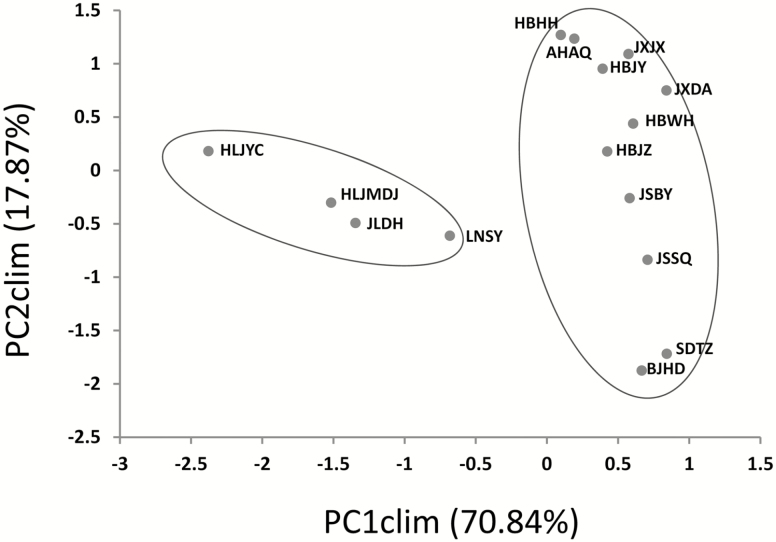
The scatterplot of the first two principal components of PCA on 22 climatic variables for the 15 sampling sites.

There was a prominent climatic heterogeneity among the sampling sites. A scatterplot of PC1clim scores against PC2clim scores showed that the four sites located at Northeast China (HLJYC, HLJMDJ, JLDH and LNSY) were separate from the other sites in climate conditions (Fig. 2). According to the PCA results on the 22 climate variables, the first two principal components explained 88.6 % of the overall climatic variations (Table 1). PC1clim, the first principal component, explained 70.8 % of the total variance and was correlated with most of climatic variables. PC2clim was highly correlated with the Max Temperature of Seed dispersal Month (bio20) and Min Temperature of Seed dispersal Month (bio21), but it only explained 17.9 % of the total variance. We took the climatic variables with high loadings (the absolute value > 0.8) for the following analyses (Table 1).

**Table 1.** Eigenvalues, percentage of variance explained by each PCA axis and the first two PC scores of a PCA of 22 climatic variables for the 15 sampling sites of *Zizania latifolia*.

	PC1clim	PC2clim
Eigenvalue	15.586	3.93
Percentage variance explained	70.844	17.865
Eigenvectors		
Annual Mean Temperature (bio1)	0.960	0.244
Mean Diurnal Range (mean of monthly (max temp − min temp)) (bio2)	−0.978	0.076
Isothermality (bio2/bio7) (* 100) (bio3)	−0.144	0.761
Temperature Seasonality (SD * 100) (bio4)	−0.963	−0.194
Max Temperature of Warmest Month (bio5)	0.931	0.274
Min Temperature of Coldest Month (bio6)	0.972	0.197
Temperature Annual Range (bio5–bio6) (bio7)	−0.969	−0.165
Mean Temperature of Wettest Quarter (bio8)	0.507	0.696
Mean Temperature of Driest Quarter (bio9)	0.954	0.200
Mean Temperature of Warmest Quarter (bio10)	0.942	0.280
Mean Temperature of Coldest Quarter (bio11)	0.965	0.226
Annual Precipitation (bio12)	0.927	−0.318
Precipitation of Wettest Month (bio13)	0.809	0.270
Precipitation of Driest Month (bio14)	0.941	−0.277
Precipitation Seasonality (coefficient of variation) (bio15)	−0.785	0.542
Precipitation of Wettest Quarter (bio16)	0.919	−0.023
Precipitation of Driest Quarter (bio17)	0.936	−0.333
Precipitation of Warmest Quarter (bio18)	0.821	0.224
Precipitation of Coldest Quarter (bio19)	0.931	−0.330
Max Temperature of Seed dispersal Month (bio20)	−0.037	0.892
Min Temperature of Seed dispersal Month (bio21)	−0.430	0.855
Precipitation of Seed dispersal Month (bio22)	−0.748	0.333

### Variations in seed characteristics

All seed characteristics varied greatly and showed significant differences among the 15 populations except for *AGL* (Table 2, and also **see****Supporting Information—Table S2**). The seed width (*SW*) ranged from 0.98 mm (HLJYC) to 1.51 mm (JXJX), which was negatively correlated with latitude (*R* = −0.827, *P* < 0.01), and the seed length (*SL*) ranged from 8.03 mm (HLJYC) to 11.30 mm (LNSY). As expected, the estimated *S*_size_ and *S*_shape_ were significantly correlated with latitude (*R*_size_ = −0.630, *R*_shape_ = 0.767, *P* < 0.05) as well. *M*_100grain_ ranged from 0.83 g (HLJYC) to 1.73 g (JXJX), with a mean value of 1.44 g. The average value of *M*_100seed_ was 1.13 g, and it ranged from 0.57 g (HJLYC) to 1.50 g (JXJX) among the populations. As a function of *M*_100grain_ and *M*_100seed_, *SPR* was significantly correlated with latitude (*R* = 0.739, *P* < 0.01), with a mean value of 0.22. The populations located at high latitudes showed relatively high *SPR*s. *WC* was also associated with latitude (*R* = 0.610, *P* < 0.05), with a mean value of 0.23 and a range from 0.09 (JSBY) to 0.46 (JLDH). The grain length (*GL*) ranged from 28.98 mm (HLJYC) to 45.84 mm (HBHH). The corresponding awn length (*AL*) ranged from 16.78 mm (HLJYC) to 28.87 mm (HBHH). *AGL* showed no significant difference among populations, with a mean value of 0.60, indicating a constant ratio between *AL* and *GL*.

**Table 2.** The measured seed traits and estimated parameters of 15 *Zizania latifolia* populations, SEs were shown in brackets. *GL*, grain length; *AL*, awn length; *AGL*, *AL*/*GL*; *SW*, seed width; *SL*, seed length; *S*_shape_, *SL*/*SW*; *S*_size_, *SL* × *SW*; *M*_100grain_, the weight of 100 grains; *M*_100seed_, the weight of 100 seeds; *SPR*, seed-pericarp ratio; *WC*, relative water content. The superior labelled letters indicate significant differences according to multiple comparisons followed by LSD tests.

Population	*GL* (mm)	*AL* (mm)	*AGL*	*SW* (mm)	*SL* (mm)	*S*_shape_	*S*_size_	*M*_100grain_ (g)	*M*_100seed_ (g)	*SPR*	*WC*
HLJYC	28.98^a^ (4.59)	16.78^a^ (3.62)	0.58 (0.05)	0.98^a^ (0.10)	8.03^a^ (1.21)	8.20^a^ (1.07)	7.86^a^ (1.80)	0.83^a^ (0.02)	0.57^a^ (0.02)	0.32^a^ (0.02)	0.25^a^ (0.03)
HLJMDJ	45.32^b^ (5.02)	26.77^b^ (3.31)	0.59 (0.02)	1.14^b^ (0.13)	10.73^b^ (1.69)	9.45^b^ (1.33)	12.19^b^ (2.75)	1.64^b^ (0.09)	1.13^b^ (0.10)	0.31^a^ (0.04)	0.31^b^ (0.04)
JLDH	41.41^c^ (7.50)	25.70^b^ (6.20)	0.62 (0.04)	1.14^b^ (0.12)	10.13^c^ (1.01)	8.92^ab^ (1.48)	11.51^bd^ (1.48)	1.42^c^ (0.09)	1.03^c^ (0.07)	0.27^b^ (0.01)	0.46^c^ (0.05)
LNSY	44.80^b^ (6.61)	27.25^b^ (5.32)	0.61 (0.04)	1.25^c^ (0.09)	11.30^d^ (0.82)	9.06^ab^ (0.77)	14.10^c^ (1.68)	1.62^b^ (0.02)	1.25^d^ (0.05)	0.23^c^ (0.02)	0.34^b^ (0.04)
BJHD	39.76^cd^ (5.27)	21.61^c^ (3.57)	0.54 (0.03)	1.07^c^ (0.14)	9.66^c^ (1.00)	9.04^a^ (1.02)	10.33^b^ (2.11)	1.17^d^ (0.02)	0.85^bc^ (0.03)	0.28^b^ (0.01)	0.26^a^ (0.03)
SDTZ	44.60^b^ (5.77)	26.69^b^ (3.97)	0.60 (0.04)	1.35^d^ (0.12)	10.16^c^ (0.76)	7.50^ac^ (0.80)	13.77^c^ (1.71)	1.45^cd^ (0.04)	1.08^bc^ (0.04)	0.25^bc^ (0.01)	0.20^a^ (0.03)
JSSQ	40.08c^d^ (8.85)	25.26^b^ (7.23)	0.63 (0.05)	1.31^cd^ (0.16)	9.03^e^ (0.99)	6.91^c^ (0.52)	11.78^b^ (2.57)	1.29^e^ (0.03)	0.90^e^ (0.05)	0.30^ab^ (0.02)	0.16^d^ (0.01)
JSBY	40.04^cd^ (5.44)	23.54^c^ (3.90)	0.59 (0.05)	1.31^cd^ (0.14)	10.45^bc^ (1.07)	7.97^a^ (0.60)	13.70^c^ (2.54)	1.52^d^ (0.08)	1.23^d^ (0.08)	0.19^d^ (0.01)	0.09^e^ (0.01)
HBWH	38.00^d^ (6.26)	23.44^c^ (4.99)	0.62 (0.04)	1.21^c^ (0.25)	8.74^e^ (0.97)	7.22^abc^ (1.34)	10.57^d^ (2.74)	1.13^f^ (0.03)	0.90^e^ (0.02)	0.20^d^ (0.01)	0.35^b^ (0.04)
AHAQ	34.10^e^ (6.87)	20.39^c^ (5.61)	0.60 (0.06)	1.41^d^ (0.15)	9.70^c^ (1.09)	6.90^c^ (0.98)	13.63^c^ (2.35)	1.31^e^ (0.03)	1.21^bd^ (0.02)	0.07^e^ (0.01)	0.14^d^ (0.02)
HBJZ	41.83^c^ (5.91)	25.92^b^ (4.37)	0.62 (0.03)	1.37^d^ (0.11)	10.67^b^ (0.83)	7.81^a^ (0.82)	14.57^c^ (1.65)	1.58^bd^ (0.05)	1.35^f^ (0.07)	0.15^f^ (0.02)	0.17^d^ (0.01)
HBJY	33.64^e^ (3.96)	19.90^c^ (3.34)	0.59 (0.04)	1.39^d^ (0.10)	10.07^c^ (0.69)	7.22^c^ (0.56)	14.04^c^ (1.69)	1.50^cd^ (0.05)	1.27^df^ (0.03)	0.16^f^ (0.00)	0.18^d^ (0.02)
HBHH	45.84^b^ (7.85)	28.87^b^ (6.17)	0.63 (0.03)	1.42^d^ (0.10)	10.24^bc^ (0.98)	7.23^c^ (0.75)	14.49^c^ (1.92)	1.63^b^ (0.03)	1.36^f^ (0.04)	0.16^f^ (0.01)	0.19^d^ (0.02)
JXDA	45.78^b^ (5.54)	28.35^b^ (5.04)	0.62 (0.05)	1.26^c^ (0.11)	9.77^c^ (0.68)	7.76^a^ (0.71)	12.30^b^ (1.58)	1.37^ce^ (0.05)	1.02^c^ (0.03)	0.26^bc^ (0.01)	0.12^f^ (0.01)
JXJX	38.33c^d^ (5.75)	23.56^c^ (4.47)	0.61 (0.04)	1.51^e^ (0.14)	9.74^c^ (0.98)	6.46^c^ (0.78)	14.68^c^ (2.33)	1.73^g^ (0.05)	1.50^g^ (0.06)	0.14^f^ (0.02)	0.16^d^ (0.02)
Mean	40.08 (5.01)	24.26 (3.42)	0.60 (0.04)	1.29 (0.13)	9.93 (0.83)	7.78(0.86)	12.80(1.85)	1.44(0.23)	1.13(0.23)	0.22(0.07)	0.23(0.03)

A PCA on the seed traits showed that the first two principal components accounted for 79.59 % of the total variance **[see****Supporting Information—Table S3****]**. PC1, which explained 50.52 % of the total variance, had its high loadings for *SW*, *S*_size_, *M*_100grain_ and *M*_100seed_; *S*_shape_ greatly determined PC2, which contributed to 29.07 % of the total variance. A scatterplot of PC1 scores against PC2 scores showed that the morphological variation of the northernmost population HLJYC is distinct **[see****Supporting Information—Fig. S2****]**.

In addition, *Z. latifolia* seeds showed a low germination percentage (11.7 %) and a strong dormancy (40.2 %) **[see****Supporting Information—Table S4**; **Fig. S3****]**. The germination percentage and dormancy varied greatly among populations, but they did not demonstrate associations with any of the other seed traits nor the climatic variables. The lowest germination percentage was observed in the JXDA population (0.5 %) along with the highest dormancy (72.0 %); the highest germination percentage could reach to 30.5 % (AHAQ) with a relatively low dormancy (19.5 %). The seeds from the HLJYC, JLDH, LNSY, SDTZ, JSBY and JXJX populations all showed relatively low germination percentages, but the dormancy differed dramatically among these populations **[see****Supporting Information—Table S4****]**. Notably, the northernmost population, HLJYC, showed an extremely high mortality (95.0 %).

### Correlation between the climatic variables and seed characteristics

**Figure 3.**
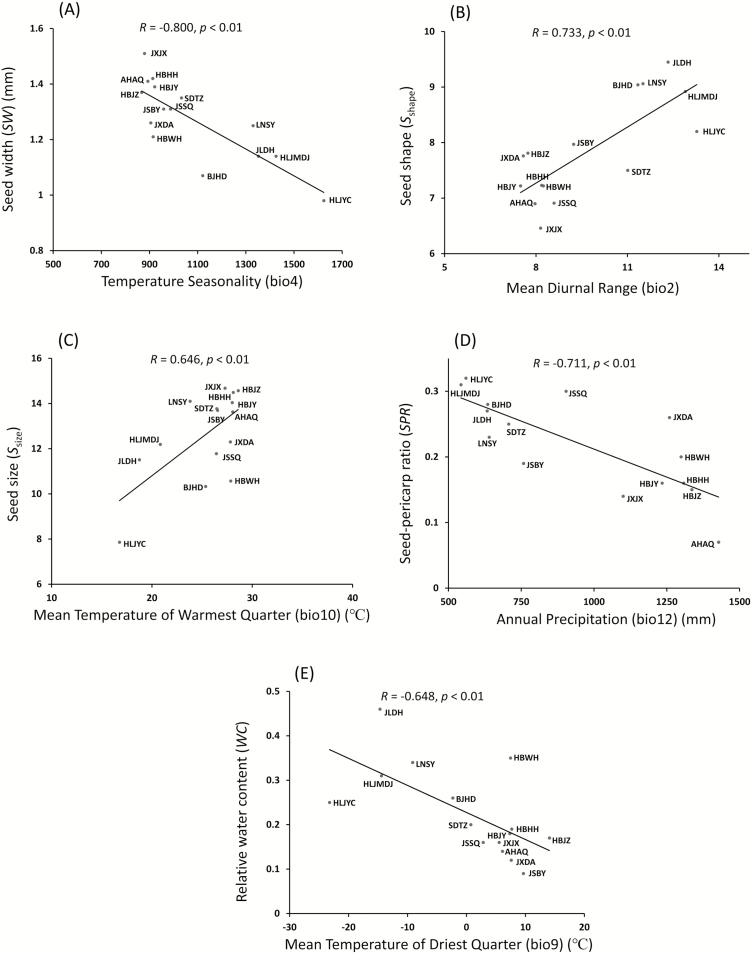
The variations of seed characteristics were significantly correlated with the climatic variables: (A) seed width (*SW*) vs. Temperature Seasonality (bio4); (B) seed shape (*S*_shape_) vs. Mean Diurnal Range (bio2); (C) seed size (*S*_size_) vs. Mean Temperature of Warmest Quarter (bio10); (D) seed-pericarp ratio (*SPR*) vs. Annual Precipitation (bio12); (E) relative water content (*WC*) vs. Mean Temperature of Driest Quarter (bio9).

The correlations between the seed characteristics and the first two principal climate components showed that *SW*, *S*_shape_, *S*_size_, *SPR* and *WC* were significantly correlated with PC1clim **[see****Supporting Information—Table S5****]**. Multiple regression analyses further indicated that *SW* was negatively affected by Temperature Seasonality (bio4) (*R* = −0.800, *P* < 0.01); *S*_shape_ was positively impacted by the Mean Diurnal Range (bio2) (*R* = 0.733, *P* < 0.01); *S*_size_ was significantly correlated with the Mean Temperature of Warmest Quarter (bio10) (*R* = 0.646, *P* < 0.01); *SPR* decreased with increased Annual Precipitation (bio12) (*R* = −0.711, *P* < 0.01); and *WC* was negatively correlated with the Mean Temperature of Driest Quarter (bio9) (*R* = −0.648, *P* < 0.01) (Fig. 3).

### Genetic diversity and structure

We found the sign of clonal growth in all sampled populations. However, the extents of clonality differed among populations (Table 3). Overall, 81 alleles were detected at the eight microsatellite loci. When the threshold was set to 1, 293 MLLs (genets) were detected from 443 sampled ramets. The effective number of genotypes (*G*_e_) varied from 1.33 (JXDA) to 26.47 (HLJYC), with an average value of 14.68, and the clonal richness (*R*) ranged from 0.11 to 0.93, with an average value of 0.65. In general, the *Z. latifolia* populations showed a high level of clonal diversity (mean *D* = 0.87). Three populations were found to have extensive clonal reproductions (HLJMDJ, BJHD and JXDA).

**Table 3.** Parameters of clonal diversity of 15 *Zizania latifolia* populations based on eight microsatellites. *N*, number of ramets; *G*, number of genets (MLL genotypes, threshold = 1); *G*_e_, effective number of genets; *R*, clonal richness [(*G* − 1)/(*N* − 1)]; *D*, clonal diversity; *E*, evenness.

Population	*N*	*G*	*G*_e_	*R*	*D*	*E*
HLJYC	30	28	26.471	0.931	0.995	0.945
HLJMDJ	26	10	2.522	0.360	0.628	0.252
JLDH	30	26	22.500	0.862	0.989	0.865
LNSY	30	19	12.162	0.621	0.949	0.640
BJHD	30	4	2.261	0.103	0.577	0.565
SDTZ	30	20	10.976	0.655	0.940	0.549
JSSQ	25	16	10.965	0.625	0.947	0.685
JSBY	30	27	25.000	0.897	0.993	0.926
HBWH	31	23	9.330	0.733	0.923	0.406
AHAQ	30	27	25.000	0.897	0.993	0.926
HBJZ	27	19	13.755	0.692	0.963	0.724
HBJY	32	27	23.273	0.839	0.988	0.862
HBHH	33	29	25.326	0.875	0.991	0.873
JXDA	29	4	1.333	0.107	0.259	0.333
JXJX	30	17	9.375	0.552	0.924	0.551
Mean	29.533	19.733	14.683	0.650	0.871	0.674

After removing the repeated ramets within the same MLLs, the *Z. latifolia* populations showed a relatively high genetic diversity (*H*_e_ = 0.495). Most of the fixation indices (*F*_is_) were significantly larger than 0 and ranged from −0.001 to 0.370, with a mean value of 0.160, indicating heterozygote deficits (Table 4).

**Table 4.** Parameters of genetic diversity of 15 *Zizania latifolia* populations based on eight microsatellites. Mean values are shown with the SE in brackets. *N*_g_, sample size; *N*_a_, number of different alleles; *A*_e_, number of effective alleles; *H*_o_, observed heterozygosity; *H*_e_, expected heterozygosity; *F*_is_, fixation index. *F*_is_ values significantly deviating from 0 are shown in bold font.

Population	*N*_g_	*N*_a_	*A*_e_	*H*_o_	*H*_e_	*F*_is_
HLJYC	28	3.375 (0.625)	2.238 (0.379)	0.390 (0.104)	0.447 (0.105)	**0.135 (0.102)**
HLJMDJ	10	2.625 (0.420)	2.061 (0.334)	0.381 (0.112)	0.440 (0.095)	0.115 (0.159)
JLDH	26	3.250 (0.453)	2.043 (0.150)	0.327 (0.062)	0.503 (0.035)	**0.370 (0.107)**
LNSY	19	5.000 (0.756)	3.258 (0.422)	0.475 (0.086)	0.675 (0.044)	**0.275 (0.126)**
BJHD	4	2.000 (0.267)	1.646 (0.226)	0.219 (0.074)	0.357 (0.099)	0.168 (0.175)
SDTZ	20	3.875 (0.479)	2.412 (0.398)	0.403 (0.088)	0.524 (0.071)	**0.164 (0.121)**
JSSQ	27	4.250 (0.818)	2.589 (0.542)	0.475 (0.104)	0.499 (0.104)	−0.001 (0.068)
JSBY	16	6.000 (1.000)	3.176 (0.595)	0.505 (0.087)	0.609 (0.072)	**0.139 (0.097)**
HBWH	27	3.750 (0.620)	2.412 (0.312)	0.421 (0.080)	0.531 (0.083)	**0.184 (0.089)**
AHAQ	4	4.625 (0.730)	2.396 (0.302)	0.428 (0.098)	0.528 (0.083)	**0.170 (0.122)**
HBJZ	17	3.500 (0.982)	2.599 (0.592)	0.427 (0.103)	0.471 (0.117)	0.043 (0.050)
HBJY	23	5.250 (0.881)	3.015 (0.387)	0.532 (0.110)	0.611 (0.086)	0.085 (0.123)
HBHH	19	5.000 (0.779)	2.857 (0.505)	0.470 (0.098)	0.561 (0.091)	**0.120 (0.103)**
JXDA	27	1.500 (0.327)	1.283 (0.185)	0.094 (0.066)	0.152 (0.099)	**0.294 (0.118)**
JXJX	29	3.375 (0.498)	2.375 (0.369)	0.383 (0.089)	0.511 (0.089)	**0.184 (0.134)**
Mean	19.058(0.742)	3.825 (0.198)	2.424 (0.109)	0.395 (0.024)	0.495 (0.024)	0.160 (0.029)

The genetic differentiation coefficient (*F*_st_) was 0.287 **[see****Supporting Information—Tables S6****and****S7****]**. A hierarchical AMOVA showed that 75.0 % of the total genetic variation was within populations and 18.5 % between populations (*P* < 0.01); 6.5 % of the total genetic variation was found between two regions revealed by the PCA on climatic variables (*P* < 0.01). A Mantel test showed significant evidence for IBD in *Z. latifolia* (*R*^2^ = 0.14, *P* < 0.01) **[see****Supporting Information—Fig. S4****]**.

**Figure 4.**
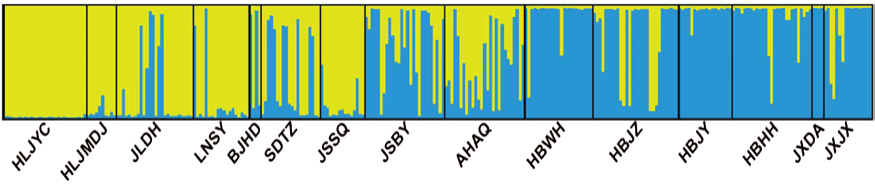
Proportions of ancestry of 15 *Zizania latifolia* populations based on *K* = 2 subdivisions. Each bar represents the proportional membership assignments of an individual.

According to the results of Bayesian assignments implemented in STRUCTURE, the most likely number of genetic groups was *K* = 2. At *K* = 2, the southern group mainly including populations from the middle reaches of the Yangtze River (HBWH, HBJZ, HBJY, HBHH, JXDA and JXJX) was divergent from the northern group in Northeast China (HLJYC, HLJMDJ, JLDH and LNSY). The remaining populations (BJHD, SDTZ, JSBY and AHAQ) had genetic consanguinity with both groups except for the population of JSSQ (Figs 1 and 4).

## Discussion

### Variations in seed characteristics along a latitudinal gradient

Latitude is a proxy for many climatic variables. For instance, the latitudinal gradient was proved to cause local environment changes that could lead to corresponding phenotypic variations in seeds (Boulli *et al.* 2001; Murray *et al.* 2003; Daws *et al.* 2006; Cochrane *et al.* 2015). It is now well established that local climatic variables can be associated with a number of seed traits, including seed size (Daws and Jensen 2011), desiccation tolerance (Daws *et al.* 2004) and longevity (Kochanek *et al.* 2010). Though the relative contribution of genetic differentiation and phenotypic plasticity on the variations of seed traits was yet to be clarified, our results indicated that *Z. latifolia* tended to produce relatively larger seeds with lower moisture content and lower investments in seed pericarp at lower latitudes. These variation trends in seed characteristics offered opportunities to screen out a batch of individuals with ideal seed traits during the neo-domestication. Multiple regression analyses further indicated that seed width (*SW*), seed size (*S*_size_), seed shape (*S*_shape_), seed-pericarp ratio (*SPR*) and relative water content (*WC*) were significantly correlated with climatic variables (Fig. 3), which could not only imply the ecological significances for variations in seed characteristics, but also could be taken as references when artificially modifying a particular seed trait.

Seed size often varies among plant species, populations and individuals (Westoby *et al.* 1992; Moles *et al.* 2005). From a macroevolutionary perspective, interspecific seed size tends to increase towards the equator, showing a latitudinal trend (Baskin and Baskin 2014). We found a similar pattern in *Z. latifolia* populations. The seed size (*S*_size_) of *Z. latifolia* was negatively correlated with latitude and positively correlated with the Mean Temperature of Warmest Quarter (bio10). Moles and Westoby (2003) suggested that the length of the growing period might positively affect seed size because longer growing seasons provide more time for carbon accumulation, which could be an explanation for this phenomenon. However, our findings were contrary to the knowledge about among-population variations in seed size that usually increased with latitude (Cochrane *et al.* 2015). The genus *Zizania* was reported to originate in North America and then dispersed into eastern Asia via the land bridge, which meant that the distribution range of *Z. latifolia* had expanded from north to south (Xu *et al.* 2008, 2010). Actually, the populations of *Z. latifolia* in the Yangtze River Basin were adjacent to the species’ southern edge. Our results may support the view that natural selection favours larger seeds towards the geographic limit (Metz *et al.* 2010). The ecological and evolutionary significance underlying the latitudinal seed size variance of *Z. latifolia* should be soundly investigated in future. Nevertheless, the populations at lower latitudes produced larger seeds, which could be predominant in grain crop domestication.

*Zizania* spp. was reported to produce recalcitrant (desiccation-sensitive) seeds (Berjak and Pammenter 2008). The typical feature for recalcitrant seeds is their vulnerability to dehydration (Dickie and Pritchard 2002; Pritchard *et al.* 2004; Daws *et al.* 2006; Berjak and Pammenter 2008). Investing the seed pericarp is a measure of the relative protection of the embryo, either from predation, pathogens or drying. The *Z. latifolia* populations at high latitudes dispersed seeds much earlier than those at low latitudes (~1–2 months earlier, field surveys), which implied that the seeds at high latitudes would remain in the soil longer before germinating. The increased *SPR* was likely to be a protective step that ensured the survival of seeds. Moreover, the significant negative correlation between seed-pericarp ratio (*SPR*) and Annual Precipitation (bio12) found in our study implied that *SPR* might be an adaptive trait to moisture availability. The clinal changed relative seed water content (*WC*) among *Z. latifolia* populations further reinforced the importance to cope with seed desiccation. Theoretically, the length of time needed to desiccate was positively associated with the relative investment in the seed pericarp and water content (Hill *et al.* 2012). In particular, the seeds collected from natural populations could hardly ensure the consistency of their initial conditions. The pattern of among-population *WC* should be fully revealed under a common garden condition in future.

A relatively low germination percentage and strong dormancy were found in *Z. latifolia*. The extents of germination percentage and dormancy differed greatly among populations, which might be attributed to maternal effects. The studies used seed collected in the wild often cannot exclude the role of maternal effects on trait variation (Donohue 2009; Cochrane *et al.* 2015). The local temperature and moisture conditions during seed development can leave a lasting impression on seed dormancy status and germination requirements (Fenner and Thompson 2005; Donohue 2009; Barua *et al.* 2012). Environmental maternal effects may evolve as a source of adaptive plasticity between generations, enhancing offspring fitness in the environment that they are likely to experience (Galloway 2005). However, we still know little about how maternal effects may vary among populations, or how important such effects will be for determining the response of recruitment to environment fluctuation. For annual crops, the production of seeds is the most important in propagation, and the dormancy of seeds is usually weak because of domestication (Hilu and Wet 1980; Doebley *et al.* 2006). However, the perennial cultivated plants could take advantages from the asexual reproduction to avoid the difficulties in seed germination due to dormancy (Glover *et al.* 2010; Gross and Olsen 2010). Unlike its annual relative *Z. palustris*, *Z. latifolia* is a perennial grass which can reproduce by tillers or rhizomes. Although the road leading to a domesticated grain crop would be rough, the perennial habit may temporarily relieve the inputs on the improvement of several domestication traits such as seed dormancy and longevity (Cox *et al.* 2006; Glover *et al.* 2010; DeHaan *et al.* 2016).

### Clonality and genetic variations in *Z. latifolia*

We did find signatures of clonal growth during our sample collections. However, the clonality of *Z. latifolia* revealed by microsatellite markers was not as strong as we had expected. The mean clonal diversity was high (*D* = 0.871). Only a few populations with relatively small population size showed significant signs of clonality. The extensive clonal growth in these populations might be the result of ‘founder effect’. For example, we consulted the locals about the population history of JXDA and found that a few ancestors had floated down from upstream and colonized here 10 years ago.

Previous phylogeographic analyses suggested that the genus *Zizania* originated in North America and then dispersed into eastern Asia via the Bering land bridge during the Tertiary (Xu *et al.* 2010). The genealogical pattern based on the nuclear Adh1a gene of *Z. latifolia* also indicated that the high-latitude populations harboured more abundant haplotypes (Xu *et al.* 2008). Xu *et al.* (2015) characterized the genetic diversity of the four species in the genus *Zizania* using three cross-specific microsatellite markers. Compared with its annual relative *Z. palustris* in North America, *Z. latifolia* showed a relatively low genetic diversity (*H*_e_ = 0.374 vs. *H*_e_ = 0.630). However, Xu *et al.* (2015) might have underestimated the genetic diversity of both species due to insufficient genetic markers. Another study addressed the genetic variations of *Z. latifolia* restricted to the middle reaches of the Yangtze River and found a high genetic diversity (*H*_e_ = 0.532) (Chen *et al.* 2012). Nevertheless, a relatively high genetic diversity (*H*_e_ = 0.497) was detected in our study. The populations located in the high-latitude areas did not show a higher genetic diversity than those in low-latitude regions. In addition, most of the fixation indices (*F*_is_) were significantly >0, indicating a heterozygote deficit that could be attributed to inbreeding or pollination between ramets.

The Bayesian assignments using STRUCTURE showed a strong genetic structure in *Z. latifolia*, which suggested limited inter-population gene flows at large scale (Fig. 4). This pattern was in line with a previous countrywide survey on the genetic structure of *Z. latifolia* (Xu *et al.* 2015). The aquatic habitat of *Z. latifolia* is discrete and patchy. The dispersal of seeds by water flow is unpredictable, and the seeds are unlikely to disperse via water currents between spatially highly isolated populations. The wind-pollinated pollens can be carried for long distances, but most effective pollination occurs locally (Willson 1983; Lu *et al.* 2005). The results of STRUCTURE demonstrated that most of the geographically intermediate populations (BJHD, SDTZ, AHAQ and JSBY) between the distinct northern and southern group had mixed genetic consanguinity (Figs 1 and 4). Notably, these populations could be connected by the Grand Canal. It is possible that gene exchanges can frequently occur via water flows in a restricted area. For example, Chen *et al.* (2012) investigated seven *Z. latifolia* populations in the middle and lower reaches of the Yangtze River and found low inter-population genetic divergence, which was attributed to the hydrological connectivity within a watershed. The physical barrier between watersheds could restrict among-population gene flows, and the genetic divergence among different water systems might be prominent (Chen *et al.* 2017). This pattern had not only been noted in the *Z. latifolia* populations in Northeast China, but also been found in another emergent aquatic grass, *Oryza rufipogon* (Wang *et al.* 2008; Fan *et al.* 2016; Chen *et al.* 2017). The inter-population genetic divergence enhances with the increase of geographical distance, which would further result in a stronger genetic structure (Wang *et al.* 2008; Zhao *et al.* 2013). Moreover, the restricted gene flow and significant habit heterogeneity were likely to lead to local adaptation that enhanced the morphological and genetic divergence. However, we could not confirm the existence of local adaptation based on current genetic data. This hypothesis still needs to be proved by corresponding common garden trials and genetic analyses (Orsini *et al.* 2013). On large scales, the clinal changed genetic consanguinity revealed by STRUCTURE and the result of Mantel test further indicated the effect of IBD played an important role in shaping the genetic structure (Fig. 1, and also **see****Supporting Information—Fig. S4**; **Table S7**).

### Implications for domestication

A good candidate for grain crop domestication would germinate rapidly when sown (low or readily breakable dormancy) and have uniform ripening (DeHaan *et al.* 2016). Large seed with shattering resistance is also of importance. Based on our results, the populations in the middle and lower reaches of the Yangtze River produce large seeds with relatively low water content and fewer investments on pericarp. Among these populations, the populations (JSBY and AHAQ) harboured genetic constitution from both northern and southern genetic groups could be ideal targets for the grain crop domestication (Huang 2009). However, if we only applied the domestication based on a few populations, it would lead to a great loss in genetic diversity due to bottleneck and founder effect. The strong genetic structure and population divergence in *Z. latifolia* also suggested even peripheral populations might contain unique genes. To fully utilize the genetic resources, the core collection of germplasm for *Z. latifolia* should be established after a thorough investigation of the morphological and genetic variations of existing natural populations.

In the meantime, seed shattering and dormancy are the first obstacles need to be overcome. Throughout history, the appearance of non-shattering grains was gradual, at least in barley, wheat and rice. In these crops, the non-shattering phenotype only occurred after an increase in grain size, a trait reflects selections on germination and production (Fuller 2007; Gross and Olsen 2010). Although it took forages a long time to breed non-shattering grains, modern domestication experiments indicated that the non-shattering phenotype could arise and increase in frequency over a short time period when subjected to strong selection (Oka and Morishima 1971; Hillman and Davis 1990). Likewise, loss of dormancy would rapidly appear under artificial selection (Hilu and Wet 1980).

In fact, the successful domestication of American wild rice could be a good example for us. In the 50 years of domestication, they not only focused on developing varieties with advanced traits such like shattering resistance, increased yield, stem sturdiness, removing seed dormancy, reduced plant height and resistance to fungal, but also generated a system of management practices (Oelke 1993). By applying the translational research from *Z. palustris* or even from *O. sativa*, there is a great chance to domesticate *Z. latifolia* to a perennial grain crop in China.

## Conclusion

There are abundant variations in the seed characteristics among *Z. latifolia* populations along a latitudinal gradient. Among the measured seed traits, *SW*, *S*_shape_, *S*_size_, *SPR* and *WC* were significantly associated with climatic variables. In general, *Z. latifolia* produces larger seeds with lower moisture content and lower investments in seed pericarp at lower latitudes. The low germination percentage and strong dormancy of seed may be potential obstacles for neo-domestication as a grain crop. A prominent genetic structure was found in *Z. latifolia* populations, which could be mainly attributed to IBD. This study provided fundamental information on morphological and genetic variations in *Z. latifolia* populations. Nevertheless, more researches on deciphering the genetic basis of important domestication traits are still needed to promote the neo-domestication of *Z. latifolia*.

## Data

The measured seed trait data and microsatellite genotype data were deposited in: https://osf.io/mdu76/.

## Sources of Funding

This study was supported by the National Natural Science Foundation of China (no. 31600293) and the China Postdoctoral Science Foundation Grant (no. 2015M571483).

## Contributions by the Authors

Y.Z., J.C. and J.R. conceived the idea and designed the research project. Y.Z., K.Z. and L.Z. collected the data. Y.Z. performed the data analysis. Y.Z. drafted the initial manuscript with contribution from J.R. and Z.S. All the authors contributed critically to the discussion and edited the manuscript before submission.

## Conflict of Interest

None declared.

## Supporting Information

The following additional information is available in the online version of this article—

**Table S1.** Geographical locations, population sizes and habitat types for 15 *Zizania latifolia* populations.

**Table S2.** Summary of one-way ANOVAs for the seed traits of *Zizania latifolia*.

**Table S3.** Eigen values, percentage of variance explained by each principal component analysis (PCA) axis and the first two PC scores of a PCA of 11 measured seed traits in *Zizania latifolia* populations.

**Table S4.** The germination percentage, dormancy and mortality for 15 *Zizania latifolia* populations.

**Table S5.** The coefficients of the correlation analyses between seed traits and the first two principal components.

**Table S6.** The parameters of genetic diversity for the eight microsatellite markers.

**Table S7.** Pairwise genetic differentiation (*F*_st_) between *Zizania latifolia* populations.

**Figure S1.** Frequency distribution of pairwise distances calculated for 443 ramets of *Zizania latifolia*.

**Figure S2.** The scatterplot of the first two axes of principal component analysis (PCA) for 11 measured seed traits of *Zizania latifolia*.

**Figure S3.** Germination percentage, dormancy and mortality of seeds for 15 *Zizania latifolia* populations across a latitudinal gradient.

**Figure S4.** The scatterplot between genetic distance and geographic distance for *Zizania latifolia* populations.

## Supplementary Material

Supplemetary Material
