# Supplementary material for: Seed characteristic variations and genetic structure of wild Zizania latifolia along a latitudinal gradient in China: implications for neo-domestication as a grain crop
Source: AoB Plants. 2018 Dec 2;10(6):ply072. doi: 10.1093/aobpla/ply072 (PMC6304442; doi:10.1093/aobpla/ply072)
Supplement: Supplemetary Material [file ply072_suppl_supplementary_material.pdf]

Table S1 Geographical locations, population sizes and habitat types for 15 *Zizania latifolia* populations

Population	Location	Longitude (°E)	Latitude (°N)	Population Size*	Habitat
**HLJYC**	Yichun, Heilongjiang	128.9	47.7	small	pond
**HLJMDJ**	Mudanjiang, Heilongjiang	129.6	44.6	medium	pond
**JLDH**	Dunhua, Jilin	128.2	43.3	large	river
**LNSY**	Shenyang, Liaoning	123.4	42.1	large	stream
**BJHD**	Beijing	116.2	40.0	medium	stream
**SDTZ**	Tengzhou, Shandong	116.8	35.1	medium	marsh
**JSSQ**	Suqian, Jiangsu	118.3	33.9	medium	river
**JSBY**	Baoying, Jiangsu	119.2	33.2	huge	lake
**HBWH**	Wuhan, Hubei	114.0	30.5	large	lake
**AHAQ**	Anqing, Anhui	117.1	30.5	huge	marsh
**HBJZ**	Jingzhou, Hubei	112.1	30.3	large	stream
**HBJY**	Jiayu, Hubei	114.0	30.0	medium	stream
**HBHH**	Honghu, Hubei	113.5	29.9	huge	lake
**JXDA**	Dean, Jiangxi	115.7	29.3	small	stream
**JXJX**	Jinxian, Jiangxi	116.2	28.3	large	lake

*Population size: small (< 500), medium (500 – 1,000), large (1,000-10,000), huge (> 10,000)

Table S2 Summary of one-way ANOVAs for the seed traits of *Zizania latifolia*

Trait	F	p
GL	19.54	< 0.01
AL	14.77	< 0.01
AGL	3.19	0.08
SW	29.89	< 0.01
SL	20.01	< 0.01
S_{shape}	9.18	< 0.01
S_{size}	23.02	< 0.01
$M_{100grain}$	64.26	< 0.01
$M_{100seed}$	55.25	< 0.01
SPR	56.13	< 0.01
WC	11.31	< 0.01

Table S3 Eigen values, percentage of variance explained by each PCA axis, and the first two PC scores of a principal components analysis (PCA) of 11 measured seed traits in *Zizania latifolia* populations

	PC1	PC2
**Eigenvalue**	5.56	3.20
**Percentage variance explained**	50.52	29.07
**Eigenvectors**		
GL	0.51	0.77
AL	0.53	0.68
AGL	0.41	0.04
SW	0.88	-0.40
SL	0.68	0.58
S_{shape}	-0.32	0.82
S_{size}	0.97	0.02
$M_{100grain}$	0.85	0.37
$M_{100seed}$	0.95	-0.01
SPR	0.69	-0.60
WC	-0.67	0.68

Table S4 The germination percentage, dormancy and mortality for 15 *Zizania latifolia* populations, standard deviations were shown in the brackets.

Population	Germination percentage (%)	Dormancy (%)	Mortality (%)
**HLJYC**	3.0 (1.4)	2.0 (2.8)	95.0 (4.2)
**HLJMDJ**	24.5 (10.6)	57.5 (7.8)	18.0 (2.8)
**JLDH**	2.5 (0.7)	31.5 (3.5)	66.0 (4.2)
**LNSY**	3.5 (2.1)	71.0 (1.4)	25.5 (3.5)
**BJHD**	25.0 (5.7)	9.0 (2.8)	66.0 (2.8)
**SDTZ**	6.0 (2.8)	33.0 (1.4)	61.0 (1.4)
**JSSQ**	13.0 (1.4)	36.0 (2.8)	51.0 (1.4)
**JSBY**	7.0 (1.4)	24.0 (2.8)	69.0 (4.2)
**HBWH**	16.0 (2.8)	64.0 (5.7)	20.0 (2.8)
**AHAQ**	30.5 (9.2)	19.5 (2.1)	50.0 (7.1)
**HBJZ**	14.0 (2.8)	21.5 (9.2)	64.5 (6.4)
**HBJY**	13.0 (4.2)	57.0 (1.4)	30.0 (5.7)
**HBHH**	11.5 (2.1)	37.5 (10.6)	51.0 (8.5)
**JXDA**	0.5 (0.7)	72.0 (2.8)	27.5 (3.5)
**JXJX**	5.5 (2.1)	67.0 (2.8)	27.5 (4.9)
**Mean**	11.7 (9.1)	40.2 (23.1)	48.1 (22.5)

Table S5 The coefficients of the correlation analyses between seed traits and the first two principal components.

	GL	***AL***	***AGL***	***SW***	***SL***	S_{shape}	S_{size}	$M_{100grain}$	$M_{100seed}$	***SPR***	***WC***
**PC1clim**	0.127	0.181	0.279	0.768**	0.129	-0.689**	0.602*	0.294	0.509	-0.704**	-0.630*
**PC2clim**	0.300	0.118	-0.509	-0.141	0.345	0.393	0.060	0.108	-0.056	0.281	0.064

** $p < 0.01$, * $p < 0.05$

Table S6 The parameters of genetic diversity for the eight microsatellite markers

Locus name	Primer sequences (5'-3')	Repeat motif	***N*f**	***N*a**	***A*e**	***H*o**	***H*e**	***F*st**
**ZM16**	CTCCTACACATCAAGGATCA<br>AAGTGATGACATTGGCACGT	(AC)	0.017	6.133	3.530	0.598	0.689	0.214
**ZM40**	CAAGCAGCAAATAGCTAGCT<br>GCCTTCATCATCTACTATAC	(CA)(TA)	0.038	3.400	2.091	0.465	0.481	0.235
**ZM24**	CTCCGCATACCACCGCATT<br>GGAACCTTGCAGAAGATGGA	(AG)	0.020	5.200	3.423	0.615	0.683	0.199
**ZM25**	GTTCTGAGTTGCAACCTGGT<br>CCCATATGTCAGCGAGACAT	(CA)(TA)	0.041	3.067	2.124	0.403	0.463	0.239
**ZM35**	GACTGATGACAACTGATGGA<br>GCACATGCTTGTGTACTTGT	(GA)	0.040	3.067	2.183	0.448	0.472	0.375
**ZM26**	CGAACCCTGCATCAAACACT<br>GATTCGGGAGTCTCCTAGTT	(AG)	0.050	2.333	1.666	0.322	0.371	0.261
**ZM36**	CACGGTCTGTATCGCTTCT<br>GAGAATGTCTAGACGAGAGT	(AG)	0.019	4.267	2.635	0.086	0.507	0.401
**ZM28**	CCCTTGCTCATGCATAGATG<br>CACCTTGCACATCAGCTCAT	(GA)(AG)	0.038	3.133	1.741	0.223	0.290	0.373
**Total**				3.825	2.424	0.395	0.495	0.287

*N*f, estimated null allele frequency; *N*a, number of different alleles; *A*e, number of effective alleles; *H*o, observed heterozygosity; *H*e, expected heterozygosity; *F*st, the fixation index.

Table S7 Pairwise genetic differentiation (*F*st) between *Z. latifolia* populations

	HLJYC	HLJMDJ	JLDH	LNSY	BJHD	SDTZ	JSBY	JSSQ	AHAQ	JXDA	JXJX	HBWH	HBJY	HBHH	HBJZ
HLJYC	0.000														
HLJMDJ	0.263	0.000													
JLDH	0.253	0.230	0.000												
LNSY	0.209	0.123	0.096	0.000											
BJHD	0.384	0.282	0.231	0.174	0.000										
SDTZ	0.195	0.229	0.119	0.140	0.249	0.000									
JSBY	0.194	0.180	0.093	0.097	0.198	0.059	0.000								
JSSQ	0.229	0.281	0.101	0.134	0.217	0.081	0.085	0.000							
AHAQ	0.236	0.160	0.111	0.121	0.169	0.106	0.064	0.105	0.000						
JXDA	0.438	0.524	0.351	0.352	0.496	0.310	0.288	0.272	0.306	0.000					
JXJX	0.275	0.236	0.200	0.160	0.249	0.145	0.111	0.124	0.092	0.217	0.000				
HBWH	0.274	0.199	0.144	0.114	0.225	0.161	0.084	0.161	0.083	0.298	0.135	0.000			
HBJY	0.265	0.259	0.201	0.166	0.227	0.148	0.105	0.168	0.120	0.207	0.123	0.107	0.000		
HBHH	0.230	0.202	0.124	0.108	0.179	0.138	0.083	0.112	0.060	0.188	0.081	0.042	0.081	0.000	
HBJZ	0.174	0.196	0.136	0.115	0.188	0.120	0.057	0.092	0.089	0.248	0.112	0.081	0.085	0.050	0.000

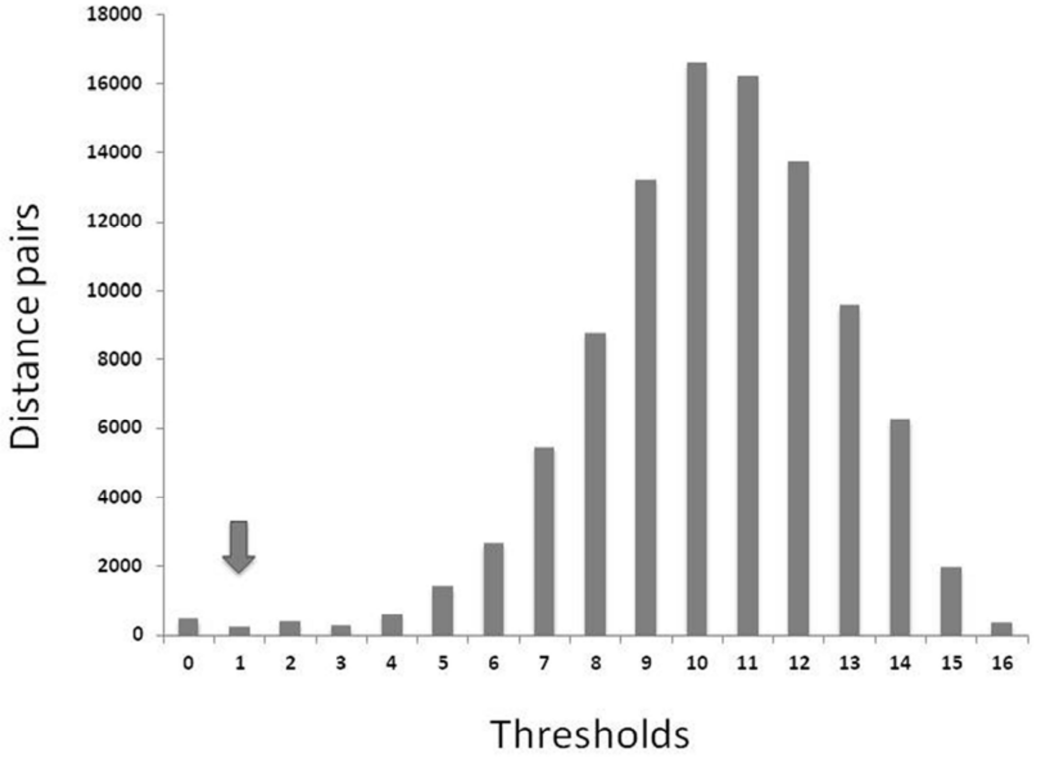

Fig. S1 Frequency distribution of pairwise distances calculated for 443 ramets of *Z. latifolia*. The arrow indicates threshold = 1

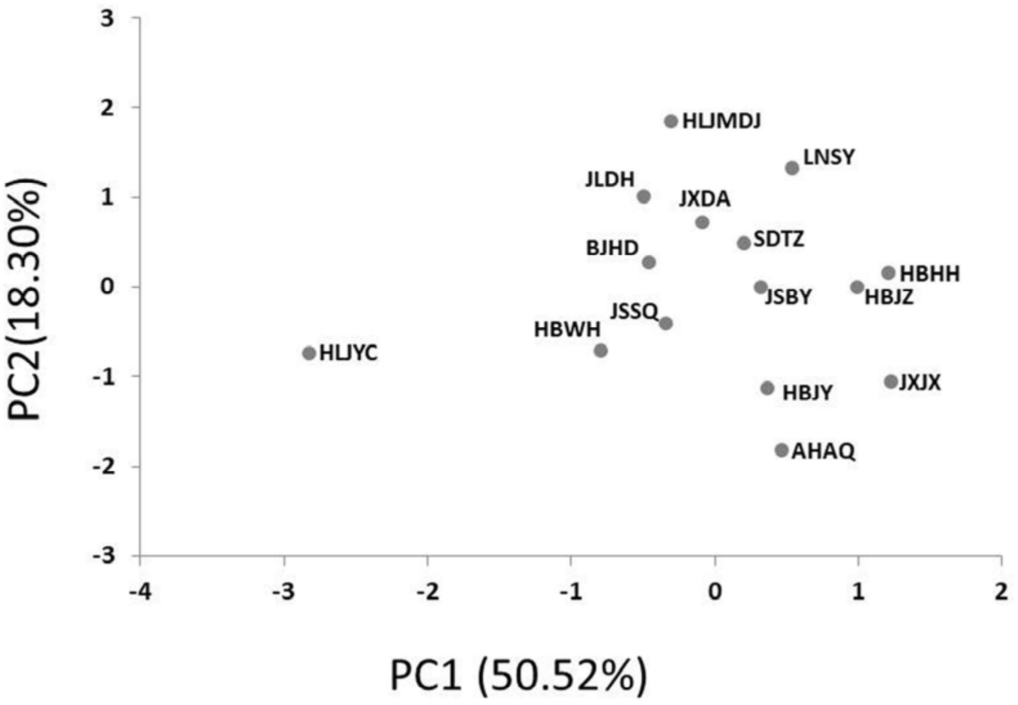

Fig. S2 The scatterplot of the first two PC axes of Principle Component Analysis (PCA) for 11 measured seed traits of *Z. latifolia*.

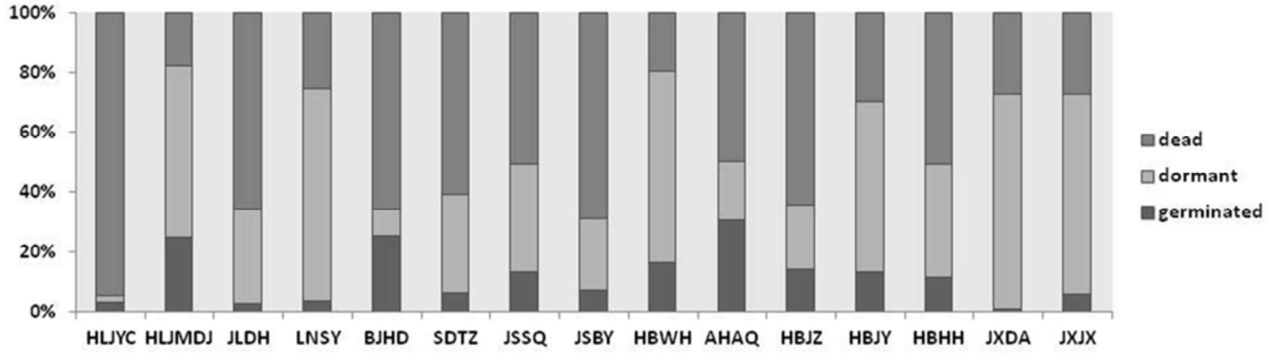

Fig. S3. Germination percentage, dormancy and mortality of seeds for 15 *Z. latifolia* populations across a latitudinal gradient.

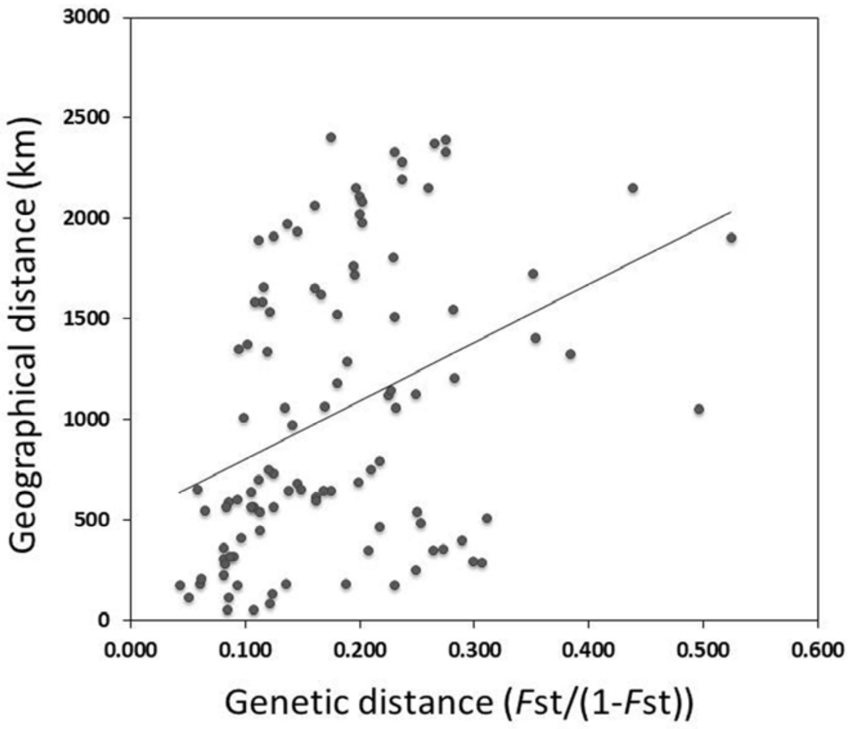

Fig. S4 The scatterplot between genetic distance and geographic distance for *Z. latifolia* populations. Genetic distance is represented by pairwise *F*st/(1–*F*st) among populations, which is correlated with geographic distance between populations. The regression line overlays the scatterplot (Mantel-test, R^2=0.14, $p < 0.01$).
